# General Nervous Manifestations in Relation to the Jaws and Teeth

**Published:** 1902-08

**Authors:** George V. I. Brown

**Affiliations:** Milwaukee, Wis.


### GENERAL NERVOUS MANIFESTATIONS IN RELATION TO THE JAWS AND TEETH.[1]

BY GEORGE V. I. BROWN, A.B., D.D.S., M.D., C.M., MILWAUKEE, WIS.

In a paper read before the Section on Neurology and Medical Jurisprudence, of this Association, in 1898, and this, the Section on Stomatology, in 1897, I outlined the theoretical propositions upon which were based methods of treatment adopted by myself in the care of nervous affections, intimately related to disturbance caused by the habit of grinding and clinching the teeth, many general, as well as local, manifestations being attributed to this etiologic factor. The term "jaw-strain" was used in the same sense as "eye-strain."

Attention was called to the fact that wherever the natural teeth remain in the mouths of patients suffering from nervous disorders, it is noticeable that the occlusal surfaces show abrasion, due to constant grinding and rubbing, the effect of extreme pressure brought to bear during paroxysmal muscular effort or long-continued, excessive pressure, a condition frequently resulting from hours of pain and suffering. This has frequent mention by writers as a marked symptom among those who are victims to the various neuroses, but in no instance do I find where an author has thought fit to reverse the order of things and make the habit of the jaws which is responsible for this condition one of the etiological factors in bringing about the disease, rather than a result, as it is generally held to be. This hyperkinetic condition of the muscles of mastication is undoubtedly due to irritation of the brain-centres

[1] Read before the Section on Stomatology, American Medical Association, Saratoga Springs, June, 1902.

governing these muscles, whether as a symptom of other neural disturbance, or *vice versa.*

Since the papers referred to were written, an almost continuous observation of such cases, and the practical application of these principles, have convinced me, more and more, that the importance of the subject is but little conceived by those who treat, as all must who treat at all, neurotics, or even victims to nervous functional diseases of lesser degree.

It is difficult to convey a due appreciation of, or, indeed, even to satisfactorily account for, general nervous manifestations, often due to dental irritation, slight though it may be locally. Yet it has occurred repeatedly in my experience with patients, who have been relieved by treatment applied solely to the etiological factor, that where, for any reason, the exciting cause was allowed to return to the original state, or the habit of irritation by grinding certain affected teeth was permitted to be resumed, the symptoms of pain, hysteria, muscular spasm, neurasthenia, or whatever they may have been, have returned at once, and again disappeared after local relief or correction has been given.

The rationale of the jaw muscle habit in its pathologic significance is thus explained. Whether central irritation be excited by some other primary disease and the peripheral irritation be a secondary result, or the local disturbance an exciter of the cortical centres, the effect as a factor in disease is manifested in several distinctly different forms, which are as follows :

1. Through the pterygoids a lateral grinding motion of the jaw of man takes place, which, if pursued at night, is easily noticeable by the grating sound, and usually attention is called to it. If, however, the masseter and temporal muscles are called into unusual activity, the result is that the jaws are firmly and tightly pressed together, without sound, and therefore often unnoticed, the pressure varying in individuals from two hundred to two hundred and seventy pounds. With the jaws closed normally and occlusion perfect, this force would be comparatively equally divided among the whole number of teeth. If, however, as usually happens in these cases, the jaws be shifted slightly to one side, a little forward or backward, then certain portions of the individual teeth are brought together, and they alone must bear this tremendous force. Ordinarily the membrane surrounding the root is capable of withstanding a considerable amount of traumatic irritation. But by continued application of this pressure, especially when weakened through other general conditions, as of circulation or otherwise, the power of resistance becomes impaired, and one of two things must result,—either a local disturbance, made manifest by elongation of the tooth, and soreness to pressure (pericementitis), a not generally serious affection, accompanied sometimes by localized pain, usually comparatively easily remedied, or, as I have believed, a direct communication of this irritation to the larger nerve-trunks, to be by them carried to the other parts.

2. In deformities of the dental arch disarrangement of the teeth must change the angle at which the stress of antagonism is applied. If the crowns or cusps occlude irregularly, whether upon the lingual, buccal, or labial aspects, the bell-shaped, or contoured, natural crowns of the teeth are crowded together, and the pressure exerted by the muscles of mastication tends to crowd out of line the teeth so situated as to be most subject to this crowding effect; the roots, being conical in form, are likewise in some degree forced away from the apical portion of the alveolar socket, and though in the apical space (so-called) allowance is made for a limited amount of movement in the line of the long axis of the root, there nevertheless results a measure of nerve-stretching, where the branches of the fifth communicate through the apical foramina.

3. Exhaustion. Eye-strain continues only in the day-time, whereas the jaw muscles work day and night, such patients being most weary upon awakening in the morning, and the pain being most marked at that hour.

4. It is only reasonable to suppose that, with the force of the jaws pressed tightly and continuously upon certain teeth, the moment such pressure is removed the immediate result would be a hyperæmia of the pericemental and other vessels about the roots; frequent repetitions of this would bring about a chronic inflammation, evinced sometimes by pyorrhœa alveolaris, or, more properly, perhaps, interstitial gingivitis, and in others a low degree of inflammation that, through peripheral excitation, gives rise to many reflex symptoms.

5. When it is remembered that the lower jaw at birth is much larger than the upper, and also that the bones of the face are much smaller proportionately, at this period, than the bones of the head, it will readily be conceded that that constant pushing upward of the wedge-shaped inferior maxillary between the bones of the superior maxillary, increasing its diameter posteriorly as well as laterally, must exercise a radical influence not only upon these, but also upon all the bones that are associated to form the nasal meati and the nasal septum which divides them. Thus the nasal, maxillary, lachrymal, ethmoid, inferior turbinated palate and sphenoid bones, which form the nasal meati, are affected in shape and relation by any force which would alter the position of their respective development; and in precisely a similar manner are the vertical plates of the ethmoid, vomer, the crests of the maxillæ, the palate bones, the rostrum of the sphenoid, and the nasal spine of the frontal bone, which combine to form the nasal septum, in all save the triangular notch of nasal cartilage, changed by any misdirection of energy which may cause a deviation in this dividing wall from its normal position or form.

Both Talbot and Kiernan make clear the fact that periods of stress, as they denominate certain stages, which markedly effect developmental and other physiological processes have much to do with health and the form as well as the character of the growth of both mind and body of the individual. There seems to be but little question as to the truth of these statements, and, if so, then why cannot stress arising at any other period from a most common cause likewise affect nerve-functions and metabolic changes.

Kiernan says, with reference to stress at the time of the eruption of the first molar, "In no small degree the struggle for existence during this period of stress centres around the development and eruption of the sixth-year molar. With the eruption of this molar, premature puberty, sexual precocity, epilepsy, gout, insanity, rheumatism, obesity, and other nutritive degeneracies may occur. All have been charged to the eruption of the sixth-year molar, whereas its irregular or difficult eruption is, like them, an expression of constitutional stress. Hygiene at this period means also constitutional mental and moral hygiene. Epilepsy, for example, is not a disease, but a symptom of weakness of certain vasomotor inhibitions. The first convulsion does not constitute epilepsy. Through a law of the nervous system, nerve action, once aroused, tends to repeat itself. In this way are established normal and abnormal habits, of which last epilepsy is one. In its early stages a habit, normal or abnormal, is easily checked. The first convulsion, therefore, could be prevented were its premonitions known. A recurrence could also be prevented were its constitutional origin recognized. Observation of the general constitution at this time, because of the irregular eruptions of the sixth-year molar, would enable the physician to nip epilepsy and many other allied conditions, in the bud. Reflex notions, however, must be flung overboard. All irritations should be removed and any constitutional irregularity treated."

If periods of stress so exactly correspond to the different stages of dental development, intra- and extra-uterine, and if, as has been shown, there are stages in the life of the individual which are marked by the onset of a predisposition to such perversions of nervous activity as are indicated by the investigations of Kiernan, corroborated by similar results reported by Talbot, then the influence of irritation of the neural mechanism of the teeth can neither be questioned nor ignored as a factor in neurology. It may be, and doubtless is, an error to suppose that the eruption of teeth at a specific period coincident with the beginning of epilepsy, or any other manifestation of psychopathic or neuropathic condition, is the cause of such disturbance; that the real difficulty attending dentition is itself brought about by the same cause as the disease; but since it is, as a rule, absolutely impossible for the therapeutist to attend prenatal conditions, the point of vital interest and of value to him is that the corelation of the mouth, teeth, and jaw physiological and pathological processes to nervous affections, in one form or another, is almost invariably the rule; that at least in a large majority of cases a measure of relief can be given, and every means of assistance certainly should be opened if the overwrought, highly irritable, and hypersensitive nerve-centres are to be given opportunity to rest and regain their normal equilibrium.

Dr. Daniel R. Brower says, "The prophylaxis of epilepsy demands much more attention than it ordinarily receives. A convulsion in the infancy of a child of neurotic inheritance is often the first manifestation of an epileptic tendency, and deserves serious attention. Children of this tendency should be relieved from severe nervous and mental strain. They should be kept from the use of alcoholics, opiates, coffee, tea, and tobacco in early age and adolescence, and from sexual irregularities and excesses. Phimosis, errors in vision, diseases and deformities of the upper air-passages, or any other abnormality, may demand attention and correction."

Surely, if such minute prophylactic measures are necessary, the ever-present, active irritation of jaw clinching and grinding cannot be overlooked, and if carefully attended to would undoubtedly assume an importance far beyond present appreciation.

Dr. Harold Moyer says, "To my mind the central point in the diagnosis of neurasthenia is the presence of the fatigue symptom."

"Rest is the sheet anchor in the treatment of neurasthenia."

Complete rest must comprehend, in the light of what I believe the future will reveal, a consideration of the special muscles now under consideration, as well as others more generally understood.

Without exhaustive reference to authorities, or quotations from the literature of pathologic neurology, which might be continued *ad infinitum*, in proof of the fact that unusual activity of the jaw muscles is an almost invariable symptom associated with disease of this nature, it seems advisable to deal directly with the evidence of practical results in the treatment of cases.

Miss C., aged twenty-five years, unmarried, anæmic, very nervous, tall and thin; affection of the throat under treatment for some time, without relief. Pain in occipital region most severe, but during the attack would spread to the frontal and other regions of the face. Nervous storms of this character were quite severe and of frequent occurrence. Correction of occlusion by grinding, particularly the tooth most effected by the habit, gave relief. Result: gained thirty-five pounds in flesh, and almost entire freedom from pain.

Mrs. M., aged about fifty years. Family of four children; history of many years of headache with short intervals of freedom from pain; attacks showed tendency to increase in severity and frequency at the time my treatment began. Support was given by the aid of an upper plate, constructed in such a manner as to relieve the anterior teeth from the strain of occlusion. Result: almost complete freedom from pain for several months past, notwithstanding severe trial by illness of one, and surgical operation upon another, of her family.

Mrs. T., mother of a family of children grown, and a grandmother. Quite thin, anæmic, and very nervous. Treatment consisted in corrected occlusion, and fastened teeth quite loose from pyorrhœa. Result: increase of ten pounds of flesh, and general improvement in health.

Miss M., aged twenty-five years. Tall, well-formed, but history of headache almost constantly for many years; sometimes very slight, but on the least unusual effort of excitement very severe; digestive disturbances quite marked; many kinds of food could not be tolerated; susceptibility to irritation very great; during the more serious periods of pain drugs were frequently used to give relief; both upper and lower arches were quite regular, except the failure to erupt a right superior bicuspid, which caused slight disarrangement of occlusion.

*Treatment.*—Habit of clinching the jaws in subconscious moments and during sleep made it necessary to insert a soft rubber pad to protect and relieve the irritating influence of direct tooth antagonism in occlusion. This was, and is, worn constantly at night. Steady pain has entirely disappeared. Recurrent attacks seem to have nearly ceased. Stomach has lost its irritability, and at this time (after many months) the patient seems quite well; general health and spirits uncommonly good.

Miss ——, aged — years. Extreme nervousness, manifested in a variety of ways; anæmic; lower arch crowded; grinding habit marked, and reported by her sister to have been unpleasantly noticeable during sleep. Appliance was adjusted to expand the arch, thus to give more room for the teeth, and in this way to remove the stress by relief of pressure from crowding, also to make occlusal irritation impossible. Result: complete disappearance of the unpleasant symptoms, full restoration to health, and very considerable increase in flesh.

After several months, the appliance was removed with the purpose in view of ultimately being able to dispense with its assistance. In a very short time there was a return to the former condition, which is again being relieved by the use of the appliance.

Miss F, aged thirty-three years, came in charge of an attendant, almost, or quite, unfit, because of mental disturbance, notwithstanding the fact that a history of the case showed a number of months treatment in a sanitarium, without relief. There were the following conditions: family history fair; a suspicion of specific history on her father's side, he having died of hemorrhage thirty years before. Her mother died of uræmia, and was a morphine subject.

*Previous History.*—She was an only child; scarlet fever at the age of five years; her ears commenced to discharge two years later, and continued discharging until two years ago. She had grip one year previous to my seeing her. She had been a mouth-breather since she could remember, and when first seen was just convalescing from a severe attack of nervous prostration. She sought relief from deafness, and to regain health sufficient for the continuance of her occupation as dressmaker.

*Treatment.*—A saddle-shaped upper arch was widened, and occlusion with the lower made as nearly perfect as practicable. Nose and ear treatment was thus facilitated, with extremely gratifying result. She was able to dismiss the attendant after about ten days' treatment. Six months later she reported herself quite well, increase in weight fifteen pounds, and able to work regularly without ill effect. This patient has since married, and seems to be entirely cured.

Mr. H., aged about fifty-five years. Family history not very clear. A brother's child was mentally defective. Married and had several children, all, as far as could be learned, in good health. Patient powerfully built, and, aside from the peculiar affection from which he suffered, was in good condition; a moderate drinker of beer; no intemperance so far as could be learned.

The pain had begun in the left side of the face five years before he came to me, in July, 1900. The symptoms are best described by reference to the illustration, which shows the distortion of the facial and eye muscles upon the affected side, which took place during paroxysms of pain. This came and went at intervals of about one and a half to three minutes. At the time of my first treatment, and for a few months before that date, the symptoms had extended to the throat, the hyperæsthesia making it almost impossible to swallow even liquid involving the pharyngeal muscles, and thus exciting excruciating pain. Upon examination it was found that all of the teeth of the upper jaw, from the left superior central incisor back, had been extracted in the hope of giving relief. The remaining tooth was elongated so that it extended below its next neighbor, the right superior central incisor, about one millimetre, and this notwithstanding the fact that the incisal portion showed the effect of abrasion every much, having been worn away by the grinding of the teeth of the lower jaw, which also accounted for the elongation, due to thickening of the pericementum about the apex of the root. In order to give a temporary relief, operation was performed with the surgical engine, by opening into the superior maxillary bone and severing the anterior branch of the trifacial. The pulp was removed from the left superior central incisor, and it was ground down so that it could no longer meet the teeth of the lower jaw in occlusion. All of the symptoms have disappeared, and the patient's appearance is as shown. Twice since the time of treatment he has returned with slight premonitory attacks of pain in the same region. Each time it was found that the affected incisor had worked its way down until it was again striking the lower teeth. Each time it was ground slightly, the relief being immediate and complete.

Miss F., aged thirty-five years. Referred by Dr. W. F. Malone, August, 1900. Family history unknown; height about five feet six inches; thin; anæmic. Burning sensation and hyperæsthesia of left side of tongue, which had begun about twelve months previously and had steadily increased. Six months later the same sensations were experienced in the throat, extending entirely across, and becoming so marked in severity as to threaten a complete nervous prostration. Upon examination, an ill-fitting piece of bridge-work, extending from the left superior cuspid to the first molar upon the same side, showed in a very noticeable degree the marks of the grinding habit due to evident malocclusion. Removal of the exciting cause and correction of occlusion gave relief. The patient reported in June, 1902, entirely free from the old symptoms and in good general health. Her weight, which was one hundred pounds at the time of the beginning of treatment, increased to one hundred and twenty pounds, with a complete disappearance of all nervous manifestations.

Mr. ——, aged about twenty-five years. Paralysis of the right side of the face had come on gradually, having first been noticed about seven or eight days before coming under my care. Careful examination by a neurologist failed to show a reasonable cause for such a condition. Patient's health seemed to be good; reflexes were found to be normal, and as he was not at all alarmed about his condition, there seemed to be no psychic element which could be held accountable. Examination of the eyes by an oculist failed to discover any exciting cause in that direction. Upon examination it was found that, through malformation of the jaws, only the molars met in occlusion, it being impossible for the patient to bring the anterior teeth into contact at all. In the hope that some relief might be given, the molars were ground down sufficiently to equalize the stress of occlusion. No other treatment was given. The symptoms began at once to disappear, the muscles regaining their normal usefulness in the same manner that it was lost to them.

How far it is either safe or wise to claim dental or maxillary irritation as exciting causes in such cases as the last one described I do not know. One other case of paralysis of the facial muscles, that had continued for a much longer time, the patient being a young married woman without children, and without, so far as could be learned, specific disease, in whom the grinding habit was very marked, was given a very considerable measure of relief immediately by extraction of a lower molar, which it was deemed wiser to extract than to attempt to treat under such grave conditions. Full restoration to usefulness of the muscles upon the affected side returned shortly after the alveolar socket had been healed. In another, a young lady aged about twenty-two years, there seemed to be a particularly intimate association between a paralysis of the entire right side, including the extremities, which lasted for several months, and was most puzzling to the physicians in charge, because no brain lesion could be diagnosed, although for a long time it was believed that the etiologic factor was of that nature. Her recovery, however, would seem to have disproved such a theory.

Many such cases as the one described could be cited that have come under my care during the last five or six years, and though results were not always as markedly successful as might have been desired, nearly all patients have been materially benefited, and other, more general treatment, assisted, while a very considerable number have been cured without other therapeutic measures. I am fully aware of the psychic element that is omnipresent in dealing with neurotics, and am also sufficiently impressed with the idea that one is apt to find too much in his own special field. Therefore it is my wish to emphasize, in conclusion, the following summary. The jaw habit may in some instances be an etiologic factor in functional nervous disarrangements, or more often, perhaps, only a symptom. But in the therapeusis of such diseases every method of treatment might reasonably be made more effective if its correction received due consideration. No dogmatic rules can be laid down by which ill effects of this muscle habit may be overcome. Careful study of individual characteristics requires the adoption, in different instances, of a great variety of methods, simple in themselves, yet requiring the greatest care and delicacy in order to be effective. One of the simplest and most beneficial appliances, one quite harmless and yet capable of very general and useful application, is a hard rubber plate, with soft velum rubber border extending over the occlusal surfaces of the teeth. The palatal portion gives a sense of firmness and security, and the soft rubber covering to the crowns of the teeth makes grinding or serious injury by undue irritation impossible. It need only be worn at night, and thus gives very little serious inconvenience to the patient if carefully adjusted.

A consideration of the methods of treatment would require much more than the limits of this discussion prescribe, but every one who undertakes the application of any corrective measures to the dental organs should remember that in the highly sensitive state common to these patients it is quite as easy to do harm through failure to perfect the occlusal mechanism as it is to benefit by accuracy and manipulative dexterity.

